# Hydrogel-Based Organic Subdural Electrode with High Conformability to Brain Surface

**DOI:** 10.1038/s41598-019-49772-z

**Published:** 2019-09-16

**Authors:** Shuntaro Oribe, Shotaro Yoshida, Shinya Kusama, Shin-ichiro Osawa, Atsuhiro Nakagawa, Masaki Iwasaki, Teiji Tominaga, Matsuhiko Nishizawa

**Affiliations:** 1 Department of Neurosurgery, Graduate School of Medicine, Tohoku University, 2-1 Seiryo-machi, Aoba-ku, Sendai, 980-8575 Japan; 2 Department of Finemechanics, Graduate School of Engineering, Tohoku University, 6-6-01 Aramaki-Aoba, Aoba-ku, Sendai, 980-8579 Japan; 3 Department of Neurosurgery, National Center Hospital, National Center of Neurology and Psychiatry (NCNP), 4-1-1 Ogawahigashi-cho, Kodaira-shi, Tokyo, 187-8551 Japan

**Keywords:** Biomedical materials, Neurology

## Abstract

A totally soft organic subdural electrode has been developed by embedding an array of poly(3,4-ethylenedioxythiophene)-modified carbon fabric (PEDOT-CF) into the polyvinyl alcohol (PVA) hydrogel substrate. The mesh structure of the stretchable PEDOT-CF allowed stable structural integration with the PVA substrate. The electrode performance for monitoring electrocorticography (ECoG) was evaluated in saline solution, on *ex vivo* brains, and *in vivo* animal experiments using rats and porcines. It was demonstrated that the large double-layer capacitance of the PEDOT-CF brings low impedance at the frequency of brain wave including epileptic seizures, and PVA hydrogel substrate minimized the contact impedance on the brain. The most important unique feature of the hydrogel-based ECoG electrode was its shape conformability to enable tight adhesion even to curved, grooved surface of brains by just being placed. In addition, since the hydrogel-based electrode is totally organic, the simultaneous ECoG-fMRI measurements could be conducted without image artifacts, avoiding problems induced by conventional metallic electrodes.

## Introduction

Neurophysiological monitoring has been widely used for diagnosis and treatment of neurological disorders such as epilepsy and chronic pain^1,2^. In particular, identifying pathological lesions by monitoring or mapping brain function is of great importance before and during neurosurgical operations of epilepsy or brain tumors which require surgical resections. Electrocorticography (ECoG) or intracranial electroencephalography (iEEG) are a type of neurophysiological monitoring by subdural electrodes, which are placed on the surface of the cortex, and thus superior than the scalp electroencephalography (EEG) in terms of spatial resolution and quality of signal^3,4^. High signal to noise ratio and low artifacts are particularly important in monitoring or mapping brain function. Therefore, the subdural electrode is more useful than scalp electrodes for monitoring before surgery and is expected to contribute to maximum excision of the pathological lesions and brain function preservation^5–8^.

Recently, much attention has been paid to the electro-hemodynamic coupling by simultaneous using of EEG or ECoG and fMRI for the analysis of the brain network. Especially, the relationship between EEG signals and the hemodynamics during epileptic seizure is of significance for elucidating the pathology of epilepsy^9,10^. To take into consideration of superior characteristics of ECoG to EEG, the ECoG-fMRI seems a more promising technique, but so far it has not been used extensively in clinical uses as EEG-fMRI since metal electrodes used in ECoG-fMRI could induce radio frequency currents that damage the brain tissue^11^. For this reason, no conventional ECoG electrodes are approved to be used in MRI by US Food and Drug Administration (FDA)^12^. Induction heating of the intracranial metal electrode caused by MRI is a drawback for long-term deep brain stimulation (DBS) for Parkinson’s disease^13^, although approximately 60% of DBS-indicated patients require an MRI within 5 years and 70% within 10 years after implantation of electrodes^14^. In addition to the problem of heating, contact between the electrode and the cortex is also a challenging problem. The loose contact between the cortex and the conventional ECoG electrodes made from metals such as Pt and silicone rubber or a parylene sheet^15^ leads to inaccurate signals and potential misdiagnosis. Recent ultrathin plastic film-based electronics^16–25^ dramatically improved the contact even with complex organ surfaces. However, the thin plastic films are hard to operate^26^ and their impermeability to body fluids hinders circulation of tissue fluids containing oxygen and nutrients, which is undesirable for long-term implantation. Therefore, there is a huge demand for a novel subdural electrode set, which is totally organic, shape-conformable, easy-to-handle, and permeable to body fluids.

**Figure 1:**
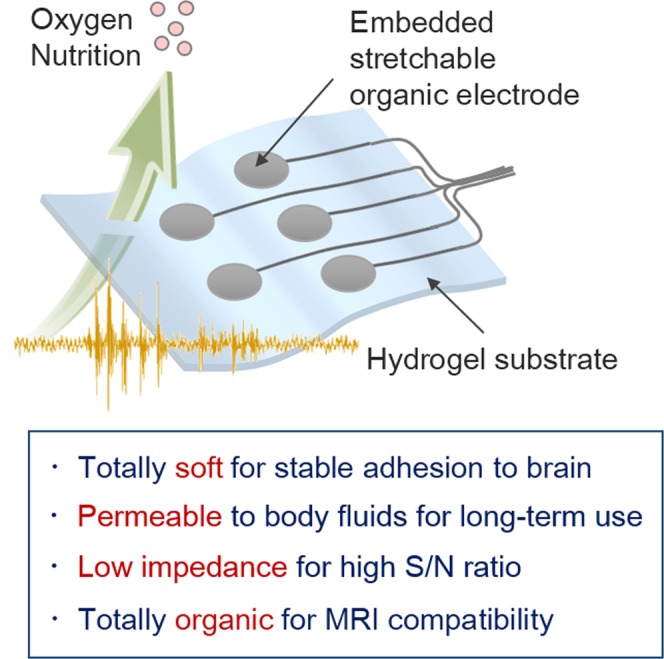
Concept and advantages of the hydrogel-based organic subdural electrode.

Here, we develop an organic ECoG electrode (Fig. 1) composed of a hydrogel substrate and a patterned stretchable carbon fabric (CF). The CF reduces the risk of generation of image artifacts and heat during MRI owing to its relatively low susceptibility and high resistivity compared to metal electrodes, and is modified with poly(3,4-ethylenedioxythiophene) (PEDOT) for better impedance characteristic in the frequency range of brain waves. Hydrogel has several advantages as a substrate material: the comparable stiffness to that of living tissues (a few ~ tens kPa)^27^, the superior operability, adhesiveness to the brain surface, and the permeability to body fluids. Taken together, the PEDOT-CF/hydrogel subdural electrode enables monitoring ECoG with a high spatial resolution and offers a solution to obtain clearer MRI images without image artifacts.

## Results and Discussion

### Integration of electrode array and hydrogel substrate

**Figure 2:**
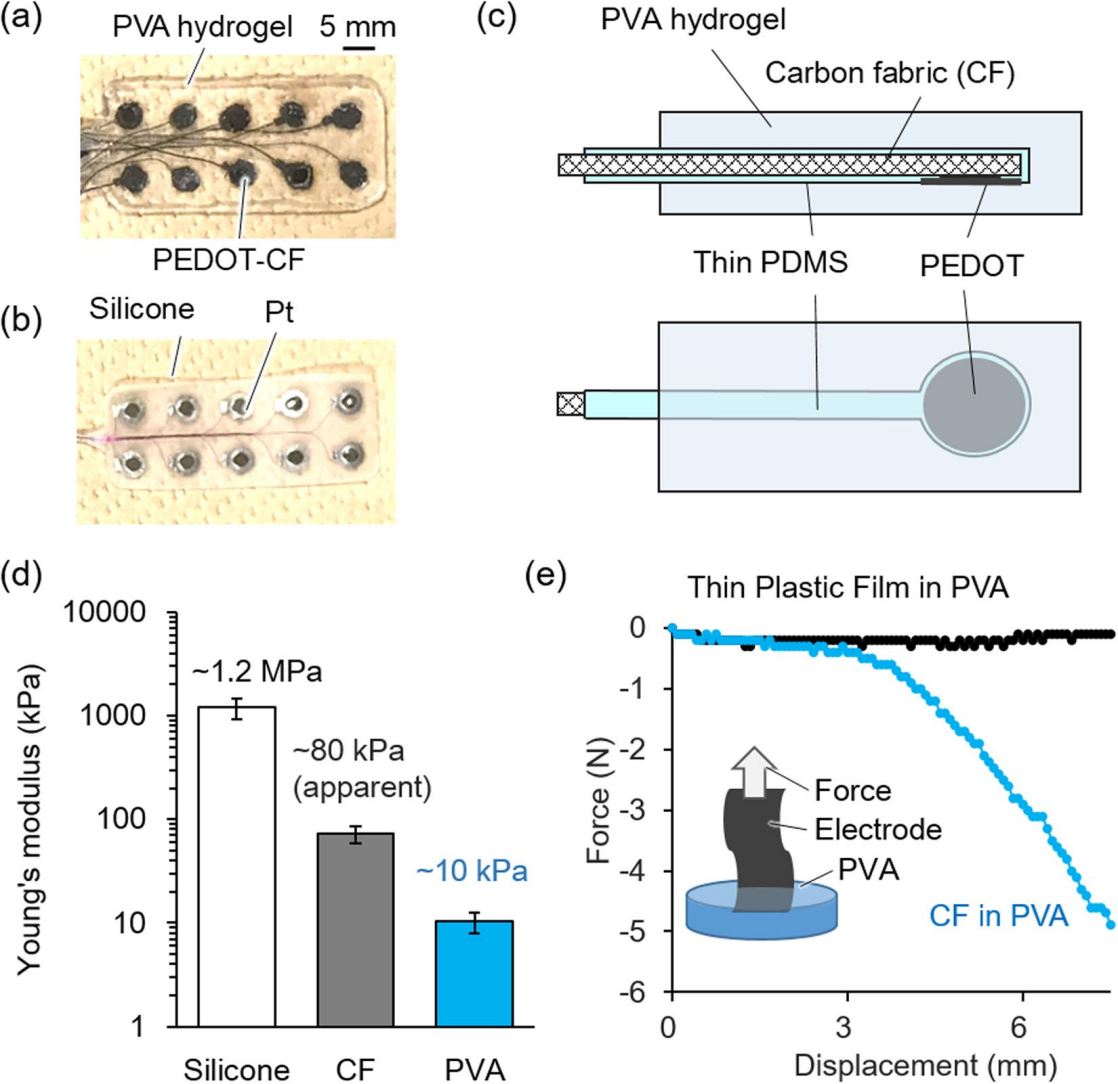
Photographs of a hydrogel-based electrode (**a**) and a conventional electrode (**b**). (**c**) The cross-sectional view (top) and top-view (bottom) of the hydrogel-based electrode. (**d**) Stiffness of silicone, CF and PVA measured by a tensile test at tensile rate of 10 mm/min (n = 3, mean ± standard deviation). Note that the stiffness of the CF shown in the paper was an apparent value since the CF was a woven mesh of carbon microfibers that expressed pantyhose-like structural stretchability. (**e**) Tensile force measured during the 8.5 mm/min pulling of a thin plastic film and CF partly embedded in PVA independently.

Figure 2a,b show the hydrogel-based organic electrode and a commercially available ECoG electrode that is made from a silicone substrate and Pt conductors. The configuration of the present hydrogel-based electrodes, in which the array of conductors is fully embedded in a hydrogel substrate, is illustrated in Fig. 2c as its cross sectional and top view. This special configuration is essentially different from the conventional coating or lamination of hydrogel onto a plastic or rubber-based electrode. Owing to the nature of hydrogel substrate, the present electrode is totally soft similarly to the living tissues and adhesive even to ridges and grooves of the brain. A carbon fabric (CF) (0.3 mm thick) was cut to be a circular electrode (3 mm in diam.) and its backside of the circle and the lead wire were insulated by coating with thin PDMS (~0.1 mm thick), followed by electropolymerization^28^ of PEDOT (2 C/cm^2^) at the exposed frontside of the circular part of the CF. The conducting polymer PEDOT has better impedance characteristic at the frequency of brain waves than the conventional metal electrodes, and is superior to other conducting polymers like polypyrrole in terms of chemical stability and electrical properties^29–31^. The array of CF was embedded within a hydrogel substrate during the gelation. Polyvinyl alcohol (PVA) (1 mm thick) was chosen as the substrate material because of its size invariance during gelation which is a critically important property for embedding the conductors. For example, there is a significant swelling of acrylamide-based hydrogel under photopolymerization, resulting in deformation of whole shape due to the mismatch in strain between the hydrogel and the embedded conductors. The ion-conductivity of PVA was examined to be about 90% of saline solution (Suppl. Fig. S1a), and the diffusivity of O_2_ through PVA was similar to that of saline solution (Suppl. Fig. S1b).

Development of the totally soft ECoG electrode requires the use of soft materials for both substrate and conductor. As shown in Fig. 2d, the stiffness of the PVA is ~10 kPa, which is in the same range as living tissues and also suitable for operation. Although the carbon fiber is known to be very stiff, the CF can deform due to its mesh structure with an apparent stiffness of ~80 kPa, which is an order of magnitude smaller than silicone (~1.2 MPs) and far smaller than Pt (~150 GPa). Another important consideration is their full integration to the substrate to avoid internal separation during the use. In order to test the toughness of integrations, force-displacement graphs were measured by pulling the samples partially embedded in PVA as shown in Fig. 2e. Results show the integration of CF and PVA is strong enough as to withstand 5 N or more pulling force, while a smooth polystyrene wrapping film easily came out without showing pulling force. The observed tough integration would be due to the 3D-integrated structure: penetration of PVA into the mesh structure of CF.

### Conformability to brain surface

**Figure 3:**
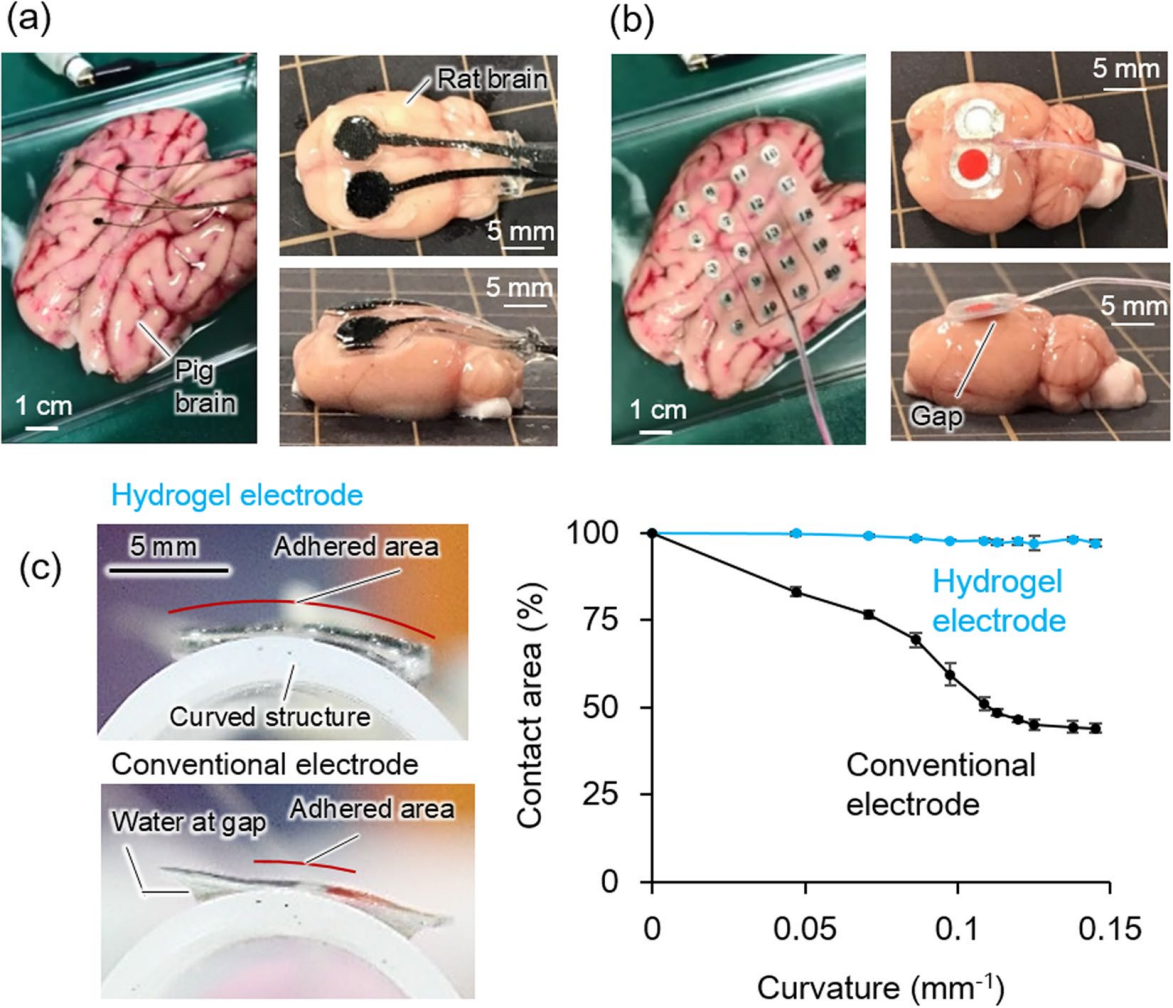
Photographs showing adherence of the hydrogel-based electrode (**a**) and a conventional electrode (**b**) placed on an extracted porcine brain and rat brain. (**c**) Side view of the electrodes placed on a curved structure wetted by water. (**d**) Contact area of the electrodes on the curved surface with various curvature (n = 3, mean ± standard deviation).

The hydrogel-based electrodes were placed on a removed porcine brain and a rat brain as shown in Fig. 3a to show their conformability to curved brain surface owing to the soft and hydrophilic nature of the hydrogel substrate which had a water content of >70%. In contrast, as shown in Fig. 3b, the commercially available silicone-based ECoG electrodes did not fully conform to the surface of brains, resulting in the gaps at their rim. In addition, silicone substrate is hydrophobic and thus slips on wet surfaces; the pressing with absorbent cotton to force better contacts is often necessary to prevent electrode displacement during ECoG measurements. The differences between the conventional electrode and the improved adherence of the hydrogel-based electrode can be clearly seen in the supplemental movies (see Movies S1 and S2). It is also important, owing to the moderate stiffness, the operability of PVA substrate (1 mm thickness) is critically superior to the ultrathin plastic films (eg., 2 μm-thick parylene) that have been considered to be used for advanced ECoG electrode^16–26^ (Movie S3).

In order to quantitatively evaluate the conformability of the hydrogel-based electrode, contact area % (the ratio of contact area in the total area of substrates) on a surface with various curvatures was observed. As can be seen in the pictures in Fig. 3c, the hydrogel-based electrode could fully adhere on a curved surface (curvature, 0.15 mm^−1^) by a direct placement, while the silicone-based conventional electrode could not fully conform to the curved surface and thus gaps were observed. The plot of contact area against the curvature depicts that almost 100% adherence of the hydrogel-based electrode can be maintained even at a curvature of 0.15 mm^−1^. In contrast, contact area of the conventional electrode decreased down to 40%, as the curvature increased. The high conformability of the hydrogel-based electrode to curved and uneven surfaces could provide a precise recording of brain wave.

### Characterization of the hydrogel-based electrode for ECoG measurement

**Figure 4:**
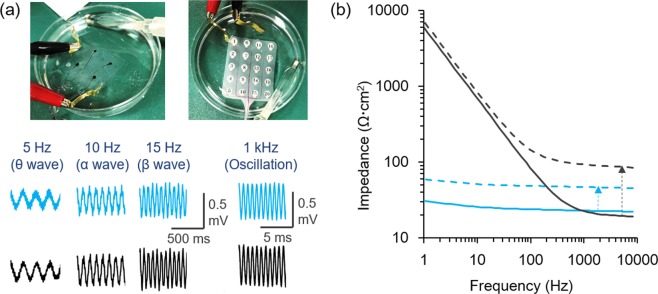
(**a**) *In vitro* recording of brain wave-like electrical signals on a saline solution. The sine waves of 0.5 mVp-p at 5 Hz, 10 Hz, 15 Hz, and 1 kHz was applied between a couple of Au electrode, and monitored by the hydrogel-based or conventional electrodes. (**b**) AC impedance spectra of the hydrogel-based PEDOT/CF electrode (blue) and the conventional intracranial electrode (black). The AC impedance was also measured with placed on a rat brain (dotted lines).

Electrical performances of the PEDOT-CF/hydrogel electrode were studied by applying brain wave-like signals using an external signal generator. Figure 4a shows that the sine waves of various frequencies ranging from 5 Hz (theta wave), 10 Hz (alpha wave), 15 Hz (beta wave), to as high as 1 kHz oscillation^3^ could be measured using the PEDOT-CF/hydrogel electrode (Fig. 4a blue lines) at a similar resolution to the conventional Pt electrodes (Fig. 4a black lines). Similar results were obtained from *ex vivo* experiments using the porcine brain instead of the saline solution (Suppl. Fig. S2).

Figure 4b shows the AC impedance spectra of the PEDOT-CF/hydrogel electrode (blue) and the conventional Pt/silicone electrode (black), recorded in the saline solution by applying AC voltage of 5 mVp-p at frequencies of 0.5 Hz~10 kHz. The impedance value of PEDOT-CF is slightly larger than the Pt at >1 kHz because of the resistivity of the CF (1 × 10^−1^ Ω cm). Particularly, the PEDOT-CF/hydrogel electrode has a lower impedance than the conventional Pt in frequency ranges lower than 1 kHz, which is the typical frequency range of brain signals^3^. This low impedance of the PEDOT-CF can be attributed to the extremely large double-layer capacitance of the fibrous conducting polymer PEDOT that has huge specific surface area^32–36^. Specifically, the double layer capacitance of PEDOT-CF and Pt were calculated to be 70 mF cm^−2^ and 0.15 mF cm^−2^, respectively, from the capacitive currents of cyclic voltammograms and the parameter fitting of AC impedance spectra (Suppl. Fig. S3). Because the potential drift (dV/dt) due to noise currents is inversely proportional to the capacitance of an electrode surface^28,37^, the PEDOT-CF is expected to exhibit the low-noise property, as demonstrated later in the animal experiments. It should also be noted that the backside and the wire of the PEDOT-CF were fully insulated, as examined by AC impedance measurements (data not shown); the impedance value of the insulated area was 1000 times higher than that of the exposed area in the frequency range of brain waves (0.1–1000 Hz), ensuring the recording takes place only at the exposed PEDOT-CF without crosstalk between electrodes. Finally, the AC impedance spectra were measured also with being placed on a rat brain (Fig. 4b, dotted lines), and the impedance values were overall increased from that of the electrodes themselves. The increase in impedance was larger for the conventional electrode as indicated by dotted arrows due to the presence of the gap between electrode and the surface of the brain. The smaller contact impedance of the hydrogel-based electrode should be an advantage for effective and precise ECoG recordings.

### MRI compatibility of the hydrogel-based electrode

**Figure 5:**
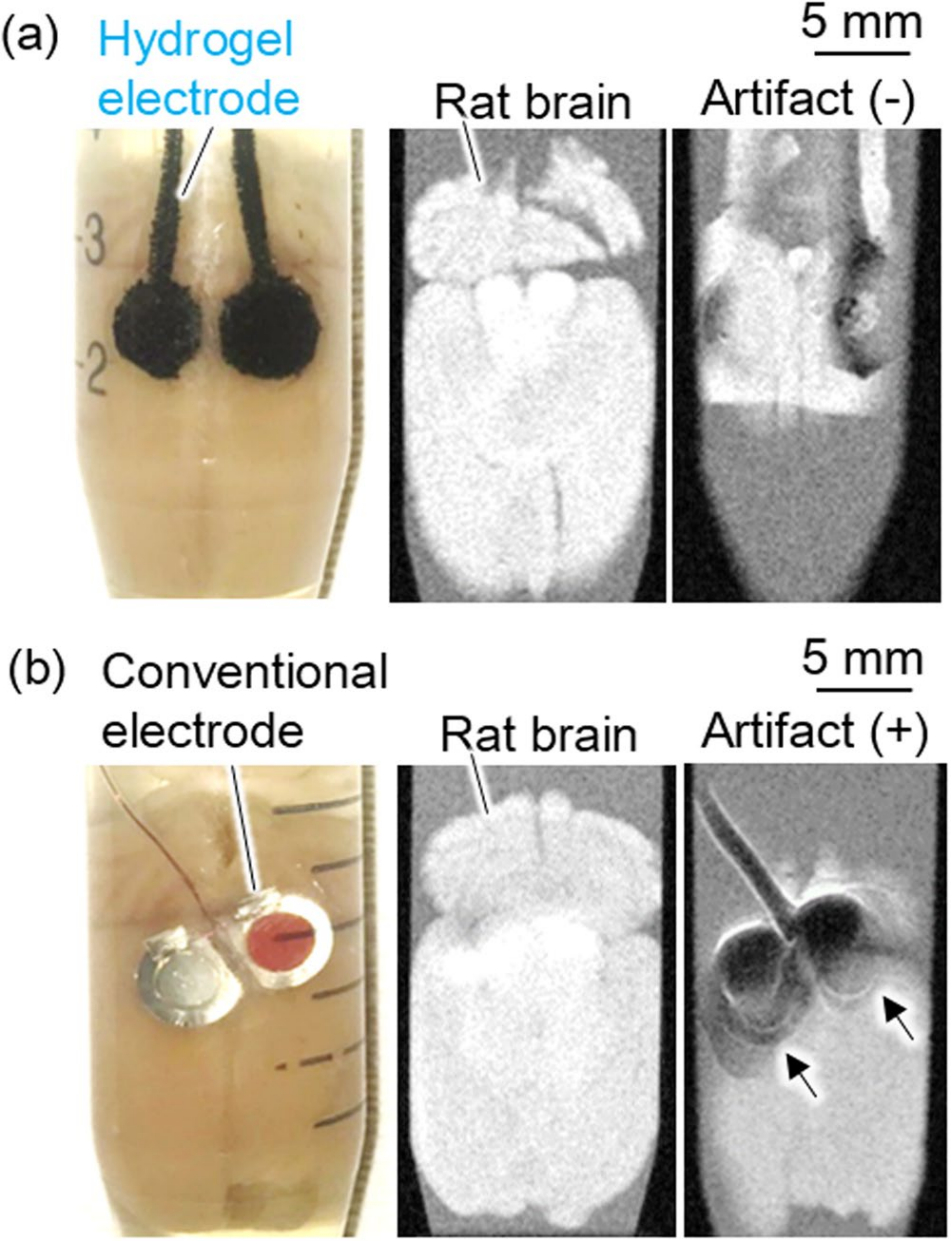
MRI measurement of (**a**) a hydrogel electrode and (**b**) a conventional electrode on *ex vivo* rat brains. Left: photographs of the electrodes on a brain placed in a tube filled with physiological saline. Middle: sliced MRI images of the specimens at a rat brain area. Right: sliced MRI images of the specimens at an electrode area showing the hydrogel electrode showed no significant artifact.

The MRI tomographic imaging was conducted with the PEDOT-CF/hydrogel electrode and the conventional Pt electrode placed on rat brains. Figure 5 shows the samples in the plastic tubes and their MRI images at two slice positions: a cross section of brain and that with electrodes placed on the cortex. Image artifacts were observed around the conventional metal electrode (black arrows) but the image from the PEDOT-CF/hydrogel electrode displayed no image artifact. This is attributed to the difference in magnetic susceptibility between metals and carbon. MRI is playing a crucial role in diagnosis of many diseases in various fields such as neurology, neurosurgery, oncology and cardiology^38–41^. EEG-fMRI and ECoG-fMRI simultaneous recording have drawn attention as advanced techniques applicable for the analysis of the brain network activity even during sleep or epileptic seizure^42–44^. However, the image artifacts and heat generation induced by metal electrodes in high magnetic field make it not practical in clinical uses. Carbon and conducting polymers are known to generate few image artifact and induction heating even in high frequency magnetic fields of MRI^45,46^. Therefore, the present PEDOT-CF/hydrogel electrode could improve the reliability and safety of ECoG-fMRI simultaneous recording for elucidating mechanisms of the brain network activity.

### *In vivo* ECoG recording with animals

**Figure 6:**
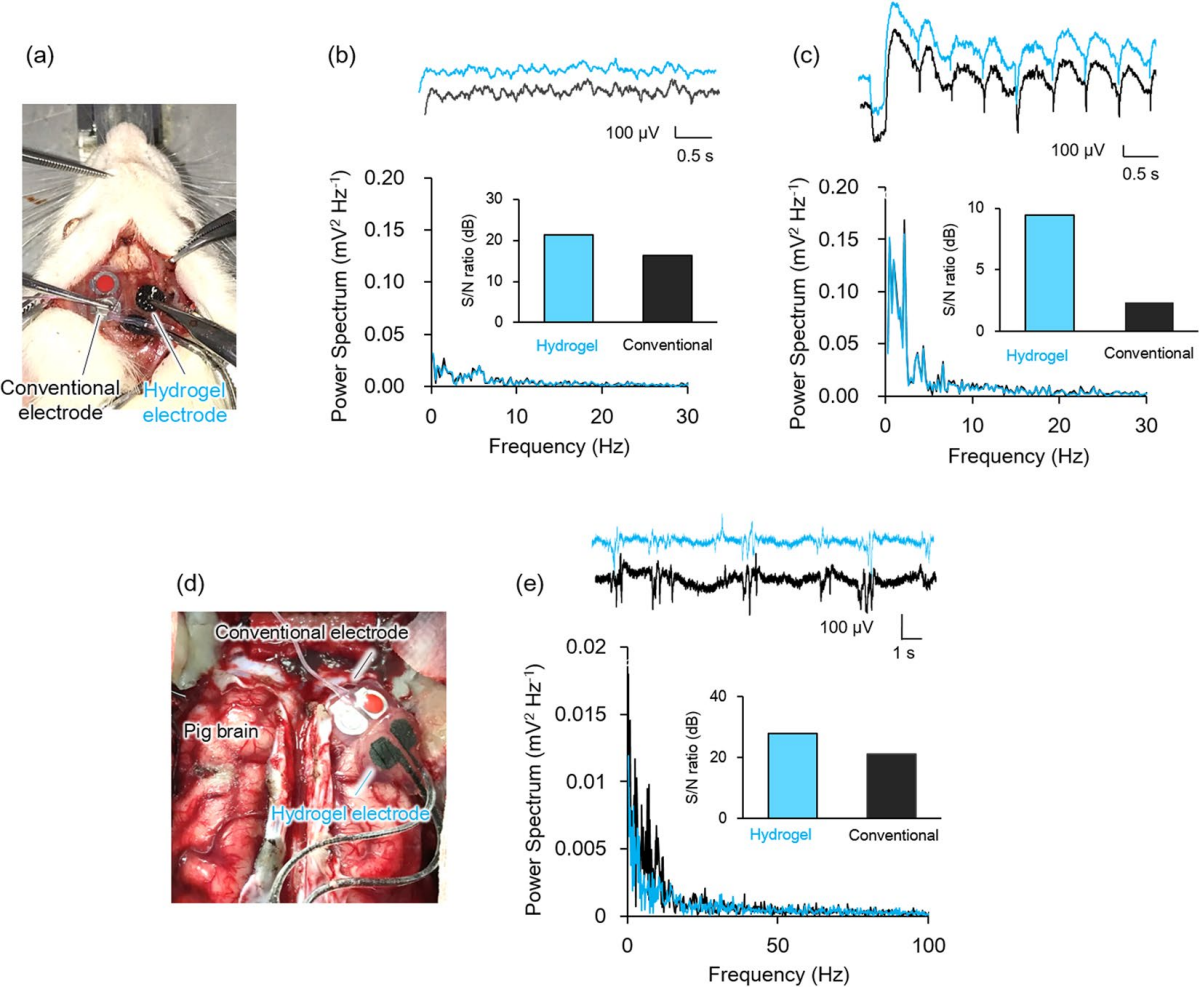
(**a**) Setting of electrodes on a rat brain for simultaneous ECoG monitoring. (**b**) The recorded brain waves of rat, the representative power spectra, and S/N ratio calculated from the spectra. (**c**) Brain waves of rat after administration of kainic acid in attempt to induce epileptic seizure, the representative power spectra, and S/N ratio calculated from the spectra. (**d**) Setting of electrodes on a porcine brain for simultaneous ECoG monitoring. (**e**) The recorded brain waves from a porcine brain, the representative power spectra, and S/N ratio calculated from the spectra.

In order to prove that the present hydrogel-based organic electrode serves as a practical ECoG electrode, *in vivo* measurements of brain waves were conducted on rats and porcines. Regarding the rat experiment, a dura mater of an anesthetized rat was partially removed by a surgery, and a surface of its cerebral cortex was exposed. As shown in Fig. 6a, the electrodes were placed on the exposed cortex, and the ECoG signals were recorded from these electrodes. *In vivo* rat brain waves were successfully acquired (Fig. 6b), of which wave forms, amplitudes and power spectrum were consistent with those reported previously from other groups^19–21^. From the power spectrum, the signal to noise ratio (S/N) of the waves were estimated using the following equation^47^,

$${\rm{S}}/{\rm{N}}[{\rm{dB}}]=10\,\log \,({{\rm{\mu }}}^{2}/{{\rm{\sigma }}}^{2})$$

where μ is mean and σ is standard deviation of the waves. The S/N ratio of the hydrogel-based electrode was higher than the one calculated from the conventional electrode, which could be owing to the larger capacitance of the PEDOT-CF (~70 mF cm^−2^) and smaller contact impedance of the shape conformable hydrogel substrate, in agreement with *in vitro* experiments (Fig. 4). Next, epileptic seizure was induced by administration of kainic acid, and brain waves were obtained after 1 h (Fig. 6c). The power spectrum of the measured brain wave showed that the epileptic brain wave could be recoded using the hydrogel-based electrode with higher S/N ratio. Finally, *in vivo* ECoG recording of porcine brain was conducted to demonstrate that the hydrogel-based electrode could also be used in a large animal model, which are more convincing toward clinical use in human. It is worthwhile to note that clear brain wave of porcine is inherently difficult to obtain^48–51^. A surface of a cerebral cortex of an anesthetized porcine was partially exposed by surgery for setting electrodes as shown in Fig. 6d. The similarity in waveforms and the power spectra suggests that hydrogel-based electrodes could also measure the porcine brain wave with higher S/N ratio (Fig. 6e). It should be noted that the conventional electrode was manually pressed on the cortex during the recording to prevent it from slipping off the brain, while the hydrogel-based electrode conformed to the cortex by simply placing on the desired position on the cortex.

As noted in the Introduction, the current application of ECoG electrodes in the treatment of epilepsy is monitoring and mapping brain functions before and during neurosurgical operations, which are the short term usage within a few weeks at most. The biochemical safety of the present hydrogel-based ECoG electrode could be ensured for the short term use, as judged from the previously reported *in-vivo* safety of PVA^52^ and PEDOT^53,54^. The live/dead cellular viability assay for three days incubation (Suppl. Fig. S5) showed nontoxicity of the PEDOT-CF/PVA electrode. The longer-term safety over months will be important for the future application such as the deep brain stimulation, and will be studied in our next investigation.

## Conclusion

In this study, we developed a totally soft organic subdural electrode by embedding PEDOT-CF into a PVA hydrogel substrate. Owing to the mechanical properties of the PVA hydrogel and PEDOT-CF, the present electrode was easy to handle and conformable to curved and grooved surfaces as demonstrated in the tight contact to *ex vivo* rat and porcine brains. Animal experiments proved that the PEDOT-CF/hydrogel electrode could be practical for ECoG recording at the frequency of brain wave (0.1 Hz~1000 Hz) by simply placing on the desired position on the cortex. In addition, the totally organic nature of the present electrode would possibly contribute to the ECoG-fMRI simultaneous measurements without image artifacts and heat generation. The hydrogel-based PEDOT-CF electrode could be a promising ECoG electrode useful for intraoperative monitoring in epilepsy surgery, and also for long-term monitoring or stimulation of the brain.

## Materials and Methods

### Materials

Poly(vinyl alcohol) (PVA, Mw~145000), and 3,4-ethylenedioxythiophene (EDOT) were purchased form Sigma-Aldrich. Dimethyl sulfoxide (DMSO), Iron(III)p-toluene sulfonate (pTs·Fe(III)), sodium chloride (NaCl), and Dulbecco’s Phosphate-Buffered Saline (PBS) were purchased from Wako Pure Chemicals. Poly(dimethylpolysiloxane) (PDMS) and curing agent (SILPOT184) were purchased form Dow Corning. Carbon fabric (CF, TCC-3250) was purchased from Toho Tenax Co. Kainic acid was purchased from TOCRIS. Brains removed from healthy rats were purchased from Japan Lamb, and porcine brains were purchased from Dard. Conventional ECoG electrode was purchased from Unique Medical. All chemicals were used as they were after purchased without further purification.

### Fabrication of the hydrogel-based electrodes

Firstly, a degassed mixture of PDMS and curing agent (volume rate 10:1) was spin-coated on a glass slide at 1000 rpm for 30 s, hardened on a 100 °C hot plate for 10 min, then another mixture of the same composition was again spin-coated on it at 600 rpm for 30 s. A piece of CF was put onto the uncured PDMS, placed on the 100 °C hot plate for 10 min, and peeled off from the glass slide with the cured PDMS sheet adhered on it. The CF was cut to be a 5-mm-diameter circle electrode and a 1 mm-width wire. A PDMS sheet that was cured after being spin-coated on a glass at 1000 rpm for 30 s was cut into a donut shape of 5 mm-outer diameter and 3 mm-inner diameter, then pasted onto the exposed side of the CF with a drop of uncured PDMS for adhesion. The wire part of the CF was connected to an insulated Cu lead wire with a heat shrink rubber tube (Sumitube C2B, Sumitomo Electric Industries), then covered with uncured PDMS followed by curing of it to be ~1 mm thick.

The exposed CF of 3-mm diameter was modified with PEDOT by electrolytic polymerization; the lead wire of the CF, an Au counter electrode, and an Ag/AgCl reference electrode were put in a solution containing 100 mM pTs·Fe(III), 50 mM EDOT and water, connected to a potentiostat (ALS/DY2323, BAS) and a coulomb meter (HF-301, Hokuto Denko Corporation). Thereafter, 1.0 V was applied until the total amount of applied charge reached 2 C/cm^2^. Finally, the PEDOT-CF electrode was washed with pure water for 1 h × 3 times (3 h in total) to remove excess pTs·Fe(III) and EDOT. The electrodes were taped on a glass slide at an interval of 5 mm, and sandwiched by another glass slide with 1 mm-thick spacer and clamps. 15 wt% PVA dissolved in a mixture of DMSO and water (mass ratio = 4:1) was poured between the glasses, then polymerized by freeze-thaw cycles (at −30 °C for 10 min and 4 °C for 10 min, each repeated three times). Fabricated hydrogel electrodes were rinsed in pure water for 1 h × 3 times, and finally kept in pure water overnight to remove cytotoxic DMSO.

### Measurement of stiffness and tensile force

Stiffness of the silicone of the conventional ECoG electrode, CF, and PVA were measured by a tensile test using a digital force gauge (ZTA-5N, IMADA) and an electromotive stage (EMX-1000N, IMADA). The bottom part of the materials were fixed to a stage and its top part was clamped to the force gauge. The force gauge was vertically moved up at a tensile rate of 10 mm/min while measuring the applied force. Young’s modulus as the index of stiffness was calculated from the early elastic region of strain-stress curves obtained from the tensile test. Note that the stiffness of the CF shown in the paper was an apparent value since the CF was a woven mesh of carbon microfibers that expressed pantyhose-like structural stretchability in addition to the elasticity of the fibers themselves. The tensile force was measured with the same setup except the specimen: a polystyrene wrapping film and CF, each embedded in a PVA hydrogel. For the tensile force measurement, the force gauge was moved up at a speed of 8.5 mm/min.

### Measurement of adhesion rate

In this study, adhesion rate was defined as the rate (%) of contact area between electrode and the surface of a curved silicone sheet (AS ONE, 2 mm-thick, cut into 3 cm × 3 cm pieces), and the rates calculated from surfaces of different curvature were investigated. To change the degree of curvature, one side of the sheet was fixed on the ground, and the other side was horizontally pushed 1 mm toward the fixed end of the sheet to deform it into an arc. The top part of the arc-shaped sheet of ~10 mm length was determined, and the curvature of the top arc of the sheet was defined as ĸ (m^−1^) = 1/R = 2 h/(r^2^ + h^2^), where R: radius of curvature, h: height of the top arc, r: half of the chord of the top arc. Hydrogel-based or conventional electrodes (width: 12 mm length: 7 mm, thickness: 1 mm) were placed on the curved surface with 30 μl water for mimicking the wet surface of *in vivo* brains, and the side view of the contacting electrodes and the surface were taken by a camera. The ratio of contact area of the electrodes to the total area of the sheet was quantified by an image analysis software (ImageJ). The adhesion rate was plotted versus degree of curvature created by incrementally pushing the silicone sheet by 1 mm.

### Measurement of electrical impedance

The hydrogel electrode and the conventional electrode were independently immersed in a physiological saline solution, and their electrical impedances at frequency ranging between 0.1–10000 Hz, 5 mV were measured by alternating current (AC) impedance measurement in which an Ag/AgCl reference electrode immersed in the saline connected to a potentiostat (ALS/DY2323, BAS). For measurement of contact impedance between the electrode and a brain, the hydrogel or conventional electrodes were independently placed on a removed rat brain that was placed in a dish filled with the saline, and AC impedance measurement was conducted using the Ag/AgCl reference electrode.

### Measurement of electrical conductivity

Physiological saline solution or PVA hydrogel containing the saline solution was separately put in a chamber of inner space: W3 × L3 × H1 mm created between two Pt electrodes with a silicone rubber spacer. AC impedance measurement at 5 mV was conducted, and the measured impedance was converted to conductivity by dividing the height (1 mm) by product of area (3 × 3 mm^2^) and the impedance.

### Measurement of oxygen diffusion

A circular Pt electrode and a circular Ag/AgCl electrode of 5 mm diameter were immersed in a physiological saline solution with an Ag/AgCl reference electrode, and chronoamperometry at −0.4 V was conducted to measure oxygen reduction currents on the Pt electrode. The Pt was covered with PVA hydrogel to show the oxygen diffusion inside the hydrogel. N_2_ bubbles were introduced in the physiological saline solution for 20 min to reduce oxygen in the solution for a negative control.

### Measurement of double layer capacitance

Electrodes were immersed in PBS, and connected to a potentiostat (ALSDY2323, BAS) with a gold counter electrode and an Ag/AgCl reference electrode. Cyclic voltammetry was performed at scanning speed 5 mV s^−1^. Double layer capacitance was calculated as division of the electric current at 0.3 V by the scanning speed. The double layer capacitance was double-checked by theoretical fitting of the electrochemical impedance spectra with an equivalent circuit model.

### Live/dead cellular viability assay

ARPE-19 cells (kindly provided by Prof. Leonard Hjelmeland at University of California, Davis, CA) were cultured in a flask containing Dulbecco’s Modified Eagle Medium supplemented with 10% fetal bovine serum and 1% Antibiotic-Antimycotic (100X, Gibco) with or without the hydrogel electrode. The cells were incubated at 37 °C in a humidified atmosphere containing 5% CO_2_ for three days. After cultivation, the cells were stained with calcein-AM, propidium iodide, and DAPI for viability assay. The stained cells were observed under fluorescent microscopy, and number of live cells (co-stained with calcein ad DAPI) and dead cells (co-stained with propidium iodide and DAPI) were counted to calculate the viability.

### *In vitro* and *ex vivo* recording of electrical signals at frequencies of brain waves

Electrical signals at frequencies of brain waves: sine waves of 5 Hz (theta wave), 10 Hz (alpha wave), 15 Hz (beta wave), and high-frequency oscillation 1 kHz at 0.5 Vp-p were generated by a function generator (WF1974, Wave Factory). For *in vitro* recording, hydrogel-based electrode and conventional electrode were independently put in a physiological saline solution (0.9% NaCl in water). For *ex vivo* recording, the electrodes were independently put on a removed porcine brain. The electrical signals at frequency of brain waves were put in the saline or porcine brain via inserted Au electrodes, and measured by the hydrogel-based or conventional electrodes connected to an amplifier (FE135 Dual Bio Amp, AD Instrument) with a recording unit (PowerLab 8/35, AD Instrument) and a software (LabChart v8, AD Instrument).

### MRI measurement

Hydrogel-based or conventional electrode were put on a removed rat brain, and put in a closed plastic tube filled with physiological saline solution. The tube was put in a MRI scanner for small animals (Bruker BioSpin ICON 1T) and images were taken as xy-plane slices from the bottom to top in the z-direction where brain and electrode existed, respectively. The images were analyzed by an image analysis software (ParaVision 6).

### *In vivo* recordings

The *in vivo* data shown in this paper are representative of different experiments from two rats and two porcines. All animal experiments were approved by the Center for Laboratory Animal Research at Tohoku University, and subject to Regulations for Animal Experiments and Related Activities at Tohoku University. Wild-type adult rats or porcines were anaesthetized with their head fixed in a stereotaxic apparatus and the dura mater was partially removed by a neurosurgion, exposing the surface of the cerebral cortex. A conventional electrode and a hydrogel electrode were placed on the exposed cortex for simultaneous ECoG recording. In the case of measurements with the rat brains, the hydrogel and conventional electrodes were placed independently on different hemisphere of the same brain fixed by tweezers since rat brains were small and relatively smooth. For the case of porcine brains, both electrodes were directly put on the same hemisphere and without additional manipulation to hold them in place. The electrodes were connected to an amplifier (FE135 Dual Bio Amp, AD Instrument) with a recording unit (PowerLab 8/35, AD Instrument) and a software (LabChart v8, AD Instrument) to obtain electrical brain activities at a sampling rate of 1 kHz with low-pass filtering at 400 Hz. Power spectrum of the brain wave was calculated by the Fast Fourier Transformation. Signal to noise ratio was calculated as described in the main text.

## Supplementary information

Supplementary Information (Fig. S1-S4)

Movie S1 Conventional electrode on an ex vivo brain

Movie S2 Hydrogel-based electrode on an ex vivo brain

Movie S3 Operability of hydrogel sheets

## Data Availability

All data generated or analyzed during this study are included in this published article (and its Supplementary Information files).
