# Supplementary material for: Hydrogel-Based Organic Subdural Electrode with High Conformability to Brain Surface
Source: Sci Rep. 2019 Sep 16;9:13379. doi: 10.1038/s41598-019-49772-z (PMC6746719; doi:10.1038/s41598-019-49772-z)
Supplement: Supplementary file 1 — Supplementary Information (Fig. S1-S4) [file 41598_2019_49772_MOESM1_ESM.pdf]

Supplementary Information

Hydrogel-Based Organic Subdural Electrode with High Conformability to Brain Surface

Shuntaro Oribe*, Shotaro Yoshida*, Shinya Kusama, Shin-ichiro Osawa, Atsuhiro Nakagawa, Masaki Iwasaki, Teiji Tominaga, Matsuhiko Nishizawa

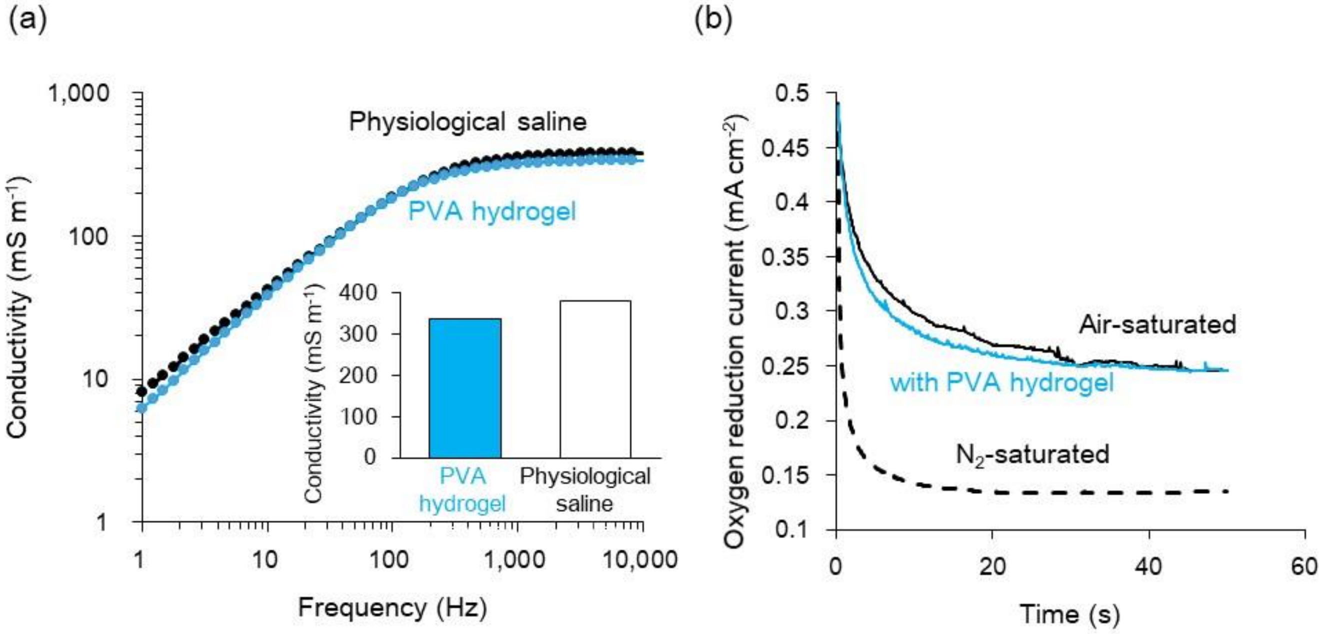

Figure S1 (a) Ionic conductivity of the PVA hydrogel containing a physiological saline solution and the solution itself measured by electrochemical impedance spectroscopy. Inset shows the conductivity of the PVA hydrogel at 1 kHz that was 88% of the physiological saline. (b) Oxygen permeability of the PVA hydrogel in a physiological saline measured by amperometry of oxygen reduction at 0V vs. Ag/AgCl with or without the hydrogel. The amperogram in an oxygen-depleted physiological saline (N_2 -saturated) is shown as a negative control.

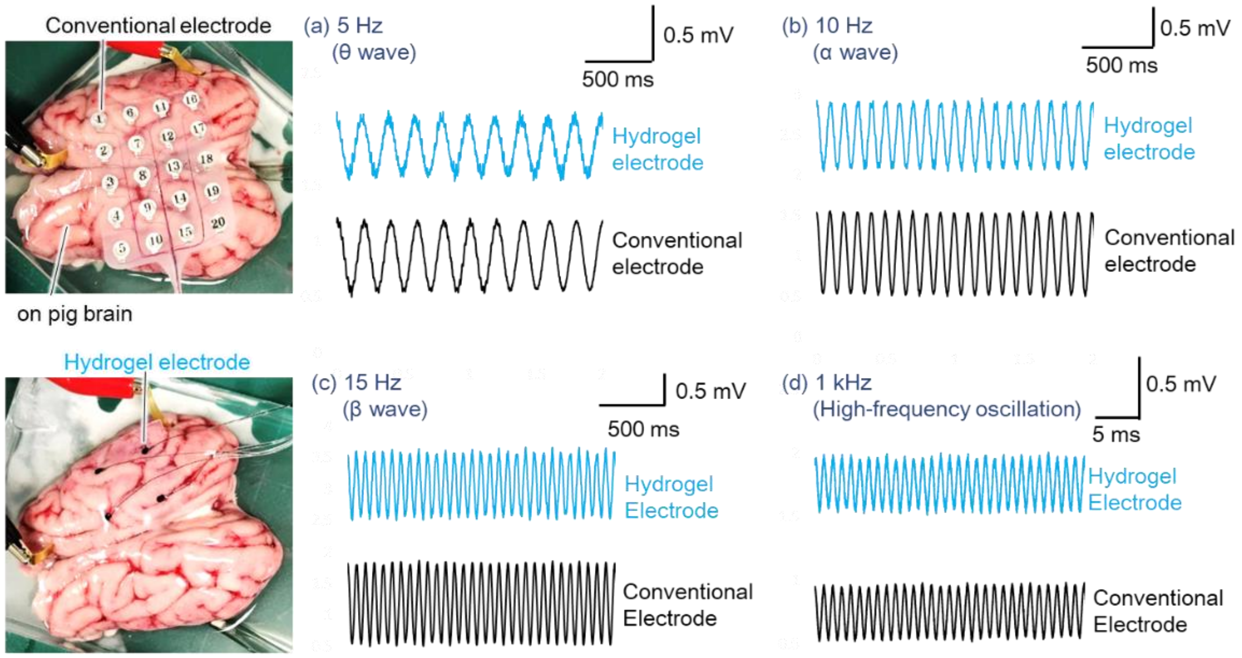

Figure S2 *Ex vivo* recording of brain wave-like electrical signals on extracted porcine brains using a conventional electrode and a hydrogel electrode. Sine waves of frequency of (a) 5 Hz, theta wave, (b) 10 Hz, alpha wave, (c) 15 Hz, beta wave, and (d) high-frequency oscillation 1 kHz were input to the porcine brain from Au electrodes and measured by the conventional or hydrogel electrodes showing similar S/N ratio. Importantly, for the conventional electrode, the electrode was pressed on to the brain to avoid slipping.

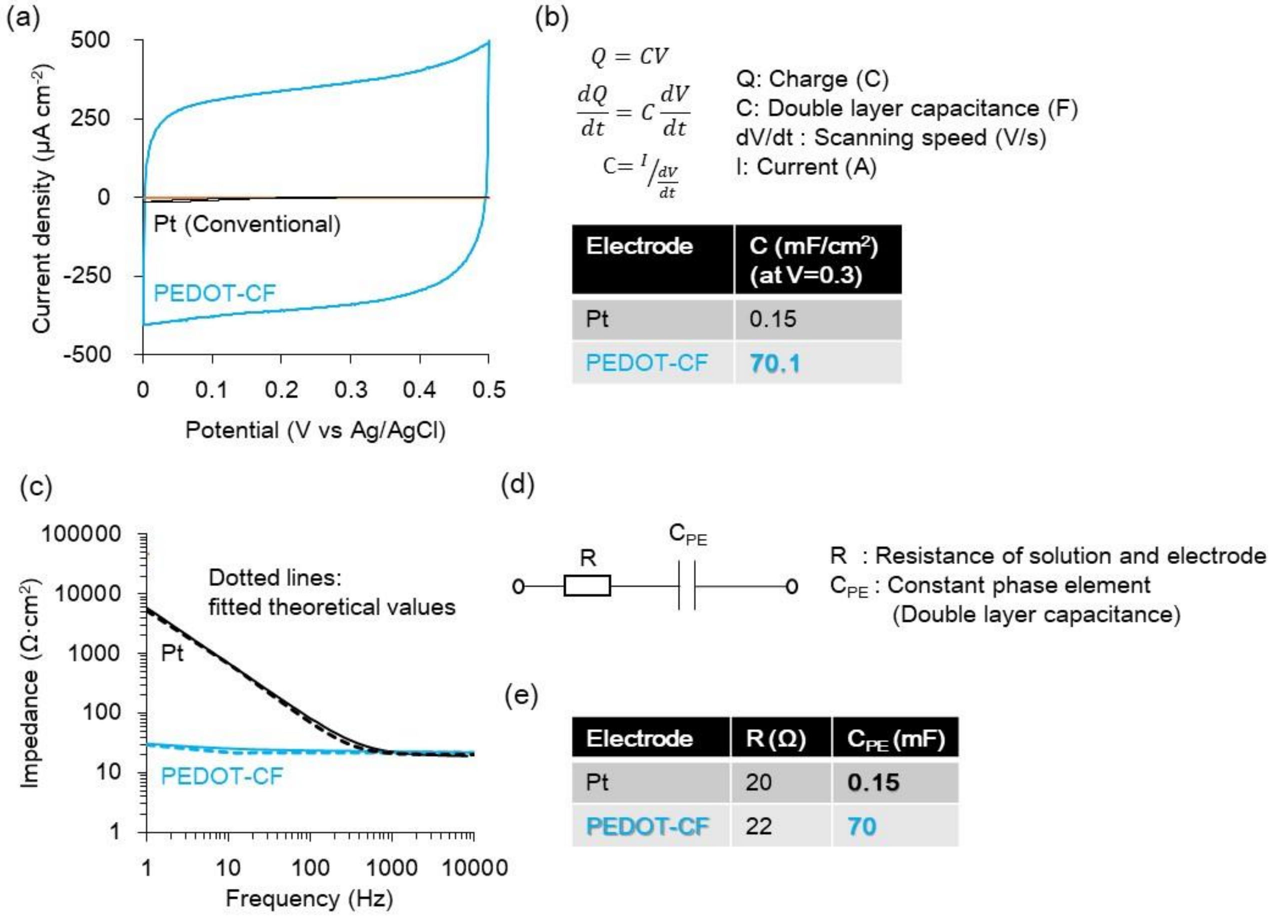

Figure S3 (a) Cyclic voltammogram of Pt and PEDOT-CF. (b) Calculation of double layer capacitance from the cyclic voltammogram. (c) Electrochemical impedance spectra of the Pt and PEDOT-CF. Solid lines represent experimental values and dotted lines represent fitted theoretical value. (d) Theoretical equivalent circuit model of the three electrodes. (e) The fitted parameters in (c) showing the larger double layer capacitance of the PEDOT-CF compared to Pt (x 470).

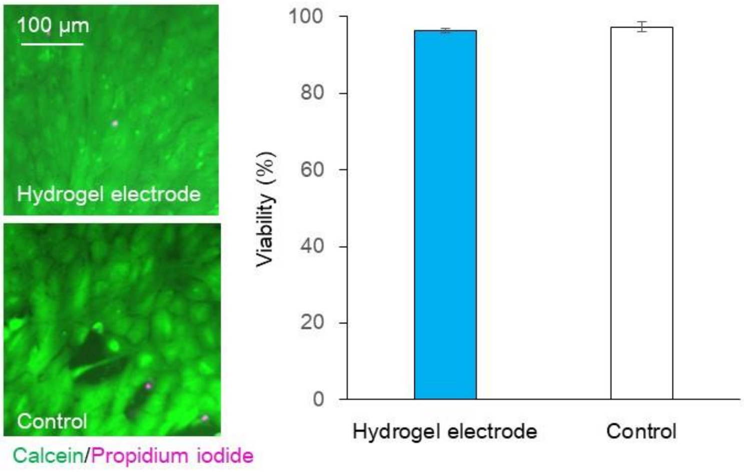

Figure S4 Biocompatibility of the hydrogel electrode. Cells were culture in a culture flask with the hydrogel electrode or without the electrode (control) for three days, and their viability was measured by a live/dead assay using calcein-AM (live) and propidium iodide (dead). (n=100, data were expressed as mean±standard deviation of 3 independent experiments)

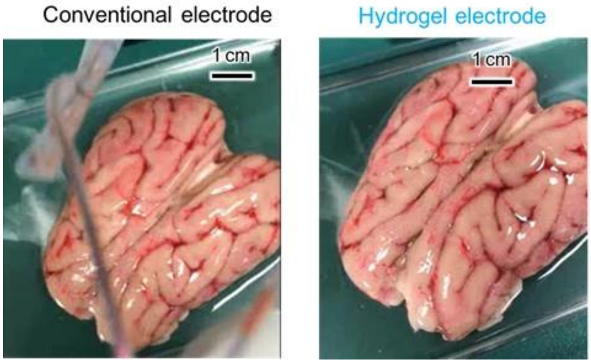

Movie S1 Low adherence of the conventional electrode to an *ex vivo* porcine brain.

Movie S2 High adherence of the hydrogel electrode to an *ex vivo* porcine brain.

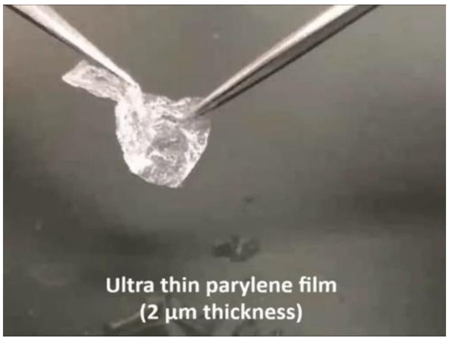

Movie S3 Operability of a hydrogel sheet superior to an ultrathin parylene film.
